# Supplementary material for: Agrochemical control of gene expression using evolved split RNA polymerase. II
Source: PeerJ. 2024 Sep 4;12:e18042. doi: 10.7717/peerj.18042 (PMC11380473; doi:10.7717/peerj.18042)
Supplement: Table S2 [file peerj-12-18042-s002.docx]

Table S2. Schematic of plasmids

| Name of plasmids | Schematic | application |
| --- | --- | --- |
| Mandi-T7-v2 | 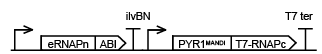 | Fig 1B, 1C, 1D, 1F, 1J |
| pCDF-T7-mcherry | 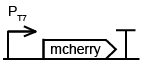 | Fig 1B, 1C, 1D |
| pJM-Mandi-T7-Ag | 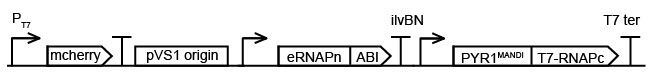 | Fig 1B |
| pCDF-T7-sfGFP | 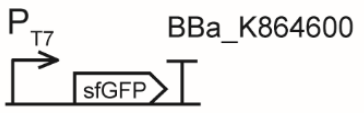 | Fig 1F |
| pCDF-T7-toehold-GFP | 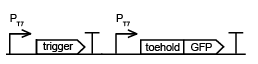 | Fig 1F, 1H |
| ZA-ZB-eRNAP2 | 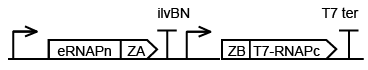 | Fig 1H |
| pJM-Mandi-T7-THS-Ag | **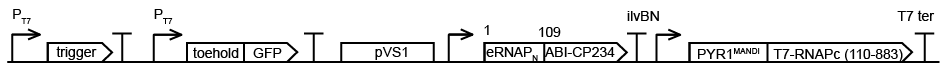** | Fig 1J |
