# Supplementary figures and images for: Agrochemical control of gene expression using evolved split RNA polymerase. II
Source: PeerJ. 2024 Sep 4;12:e18042. doi: 10.7717/peerj.18042 (PMC11380473; doi:10.7717/peerj.18042)

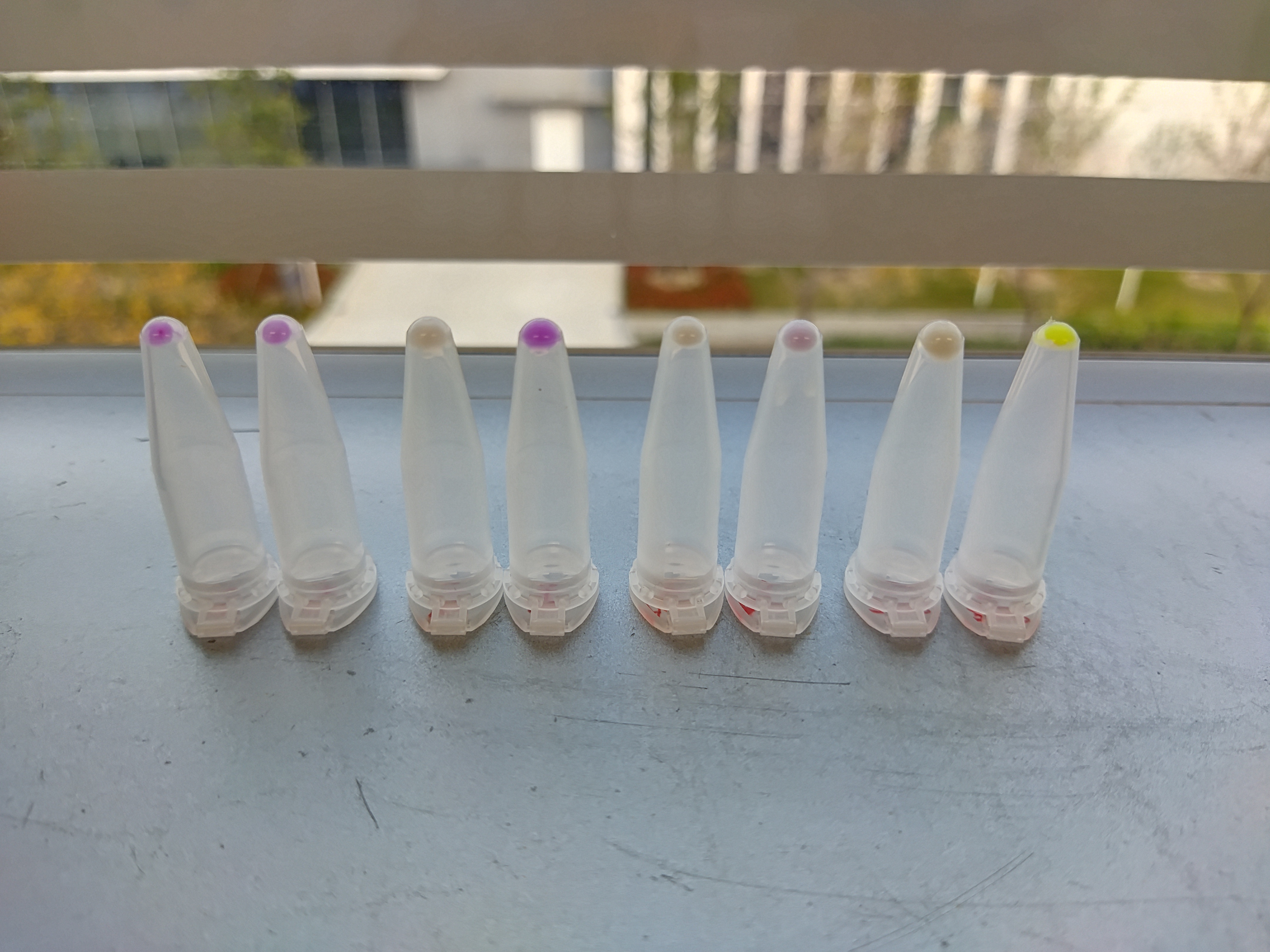

Supplement: Figure S1 — Samples were placed on the sill of a window with normal day light. +: Mandipropamid (50 μM final); -: DMSO. [file peerj-12-18042-s003.jpg]

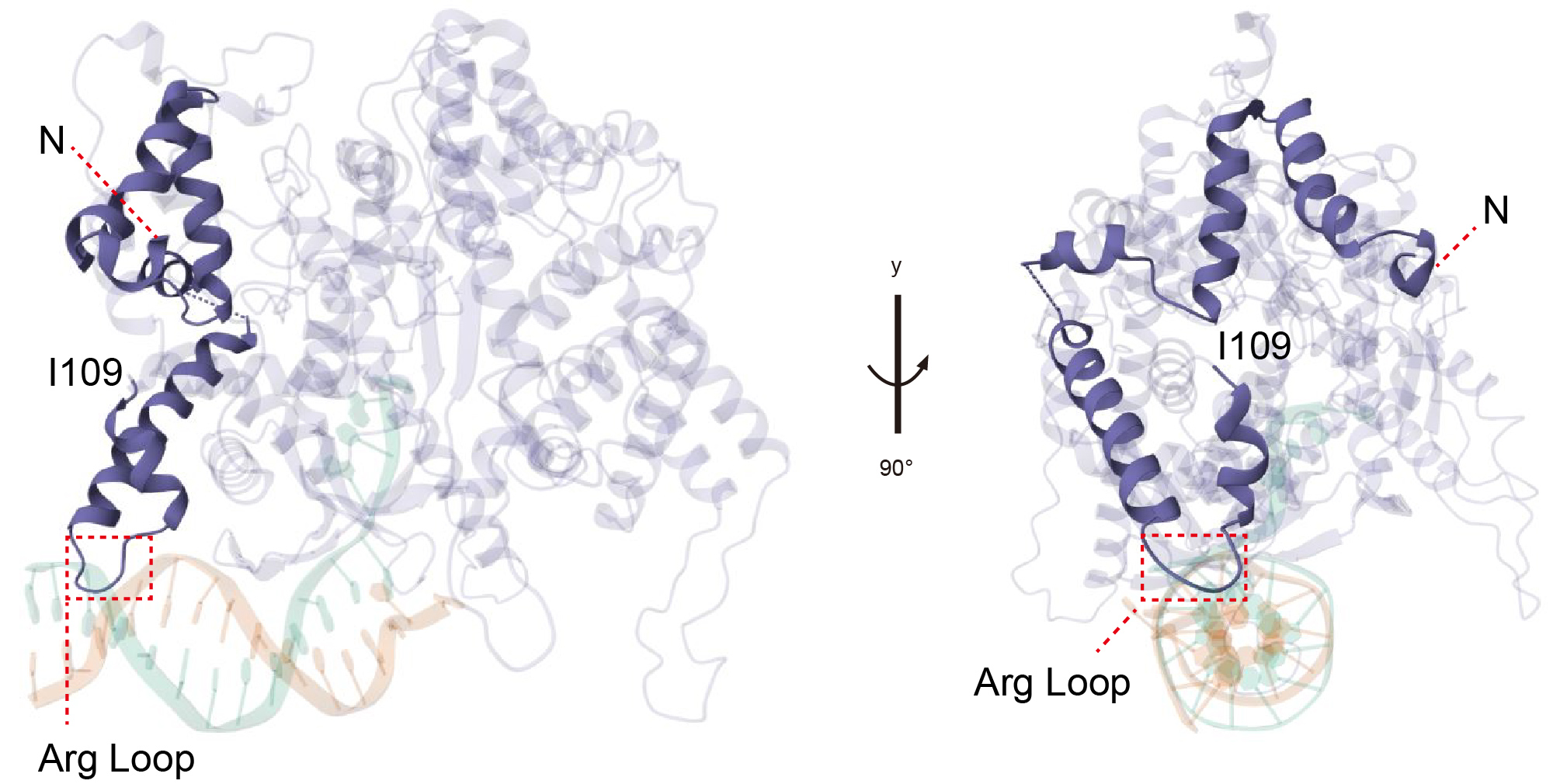

Supplement: Figure S2 — The N terminal region (1-109) of the T7 RNA polymerase was highlighted in dark purple. The Arginine loop responsible for binding the AT-rich region was encircled by a red square. The cartoon was generated from the structure of T7 RNA polymerase –T7 promoter complex (PDB ID: ICEZ). [file peerj-12-18042-s004.jpg]
